# Snakebite is Under Appreciated: Appraisal of Burden from West Africa

**DOI:** 10.1371/journal.pntd.0004088

**Published:** 2015-09-23

**Authors:** Abdulrazaq G. Habib, Andreas Kuznik, Muhammad Hamza, Maryam I. Abdullahi, Basheer A. Chedi, Jean-Philippe Chippaux, David A. Warrell

**Affiliations:** 1 College of Health of Sciences, Bayero University, Kano, Nigeria; 2 Celgene Corporation, Warren, New Jersey, United States of America; 3 Institut de Recherche pour le Développement, Cotonou, Benin Republic and Université Paris Descartes, Sorbonne Paris Cité, Faculté de Pharmacie, Paris, France; 4 Nuffield Department of Medicine, University of Oxford, Oxford, United Kingdom; University of Kelaniya, SRI LANKA

## Abstract

**Background:**

Snakebite envenoming (SBE) is a major problem in rural areas of West Africa (WA). Compared to other Neglected Tropical Diseases (NTD), the public health burden of SBE has not been well characterized. We estimated the impact of snakebite mortality and morbidity using the Disability Adjusted Life Years (DALYs) metrics for 16 countries in WA.

**Methods:**

We used the reported annual number of SB deaths and mean age at time of SB and converted these into years of life lost (YLL). Similarly, the years of life lived with disability (YLD) were estimated by multiplying the number of amputations by the respective disability weight of 0.13.

**Results:**

In WA, the annual cases of SB mortality and amputations ranged from 24 (95% Confidence Interval: 19–29) and 28 (17–48) respectively in Guinea-Bissau with the highest estimates of 1927 (1529–2333) and 2368 (1506–4043) respectively in Nigeria. We calculated that the annual DALYs associated with a SB death ranged from 1550 DALYs (95%CI: 1227–1873 DALYs) in Guinea Bissau to 124,484 DALYs (95%CI: 98,773–150,712 DALYs) in Nigeria. The annual DALYs associated with amputation for the two countries were 149 DALYs (95%CI: 91–256 DALYs) and 12,621 DALYs (95%CI: 8027–21,549 DALYs) respectively. The total burden of SBE was estimated at 319,874 DALYs (95% CI: 248,357–402,654 DALYs) in the 16 countries in WA. These estimates are similar, and in some instances even higher, than for other NTDs encountered in WA (e.g., Buruli ulcer, Echinococcosis, Intestinal Nematode Infections, Leishmaniasis, Onchocerchiasis, Trachoma and Trypanosomiasis) as reported in the Global Burden of Diseases 2010 (GBD).

**Conclusions:**

The public health burden of SBE in WA is very substantial and similar to other more widely recognized NTDs. Efforts and funding commensurate with its burden should be made available for the control of snakebite in the sub-region.

## Author Summary

Snakebite envenoming (SBE) is a major problem in rural West Africa (WA). However, despite the high incidence of SBE in this region, government funding for the prevention or treatment of SBE is generally limited. In this analysis, we attempted to estimate how the public health burden of SBE compares to other more widely recognized Neglected Tropical Diseases (NTD). To this end, we estimated the impact of SBE mortality and morbidity based on the methodology outlined in the global burden of disease and reported our results in Disability Adjusted Life Years (DALYs) for 16 countries in WA. We calculated the total burden of SBE in WA at 320,000 DALYs (95% CI: 248,000–403,000 DALYs) per year with the least and highest burdens in Guinea-Bissau and Nigeria accounting for 0.5% and 43%, respectively. The vast majority of the public health burden (91%) is attributed to early mortality. We conclude that the public health burden of SBE in WA is substantial and similar to, and in some cases even exceeds, other more widely recognized NTDs such as Buruli ulcer, Echinococcosis, Intestinal Nematode Infections, Leishmaniasis, Onchocerchiasis, Trachoma and Trypanosomiasis. Efforts and funding commensurate with its public health burden should be made available for the control of snakebite.

## Introduction

Snakebite envenoming (SBE) is a major public health problem among communities of the savanna region of West Africa, notably in Benin, Burkina-Faso, Cameroon, Chad, Ghana, Nigeria, Senegal and Togo [1,2,3,4]. The precise incidence of snakebite is difficult to determine and is often grossly underestimated. An early estimate in northeastern Nigeria reported a bite incidence of 500 per 100,000 population per year with a 12–20% natural mortality, with carpet vipers (*Echis ocellatus*) accounting for at least 66% [5]. However, this study probably exaggerated the overall incidence by extrapolating from data in selected areas notorious for their incidence of snakebites. Up to 10% of hospital beds may be occupied by SBE patients in certain areas of the country. A recent global reappraisal estimated 10,001 to 100,000 snakebite envenomings with an incidence of 8.9–93.3/100,000 persons per year with an estimated 1,001 to 10,000 deaths and a mortality rate of 0.5–5.9/100,000 persons per year occurring in the West African sub-region [6]. A more recent study estimated over 314, 000 bites, 7300 deaths and nearly 6000 amputations occurring annually in sub-Saharan Africa (SSA) [4]. As a condition affecting poor vulnerable rural dwellers, it is not only a major health problem but also a major impediment to economic prosperity from loss of income following initial incapacitation, hospitalization, long-term disabilities and premature deaths [7]. It is preventable and treatable with antivenom which has been shown to be cost effective [8]. In this analysis, we estimated the impact of snakebite mortality and morbidity using the Disability Adjusted Life Years (DALYs) metrics for 16 countries in Western Africa (WA). This will allow for comparison to other diseases as well as guide prioritization of resource allocation.

## Methods

From the most recent reliable literature available, projected annual burden of SBE in Sub-Saharan Africa was derived using a meta-analytic approach which has been described in detail elsewhere [4]. In summary, SBE data was obtained using a meta-analytic approach based on indexed, non-indexed or grey literature and conference proceedings over the past 40 years. Studies included in the analysis were categorized based on type of survey (national, household and hospital studies) and location whether conducted in urban or rural areas; with the latter representing 95% of envenoming. The pooled incidence rates, amputation rates and mortality rates were obtained and applied to the population size to derive the mortality and amputation estimates (see S1 Annex) [4]. For each country in Western Africa the annual number of snakebite deaths and mean age at time of envenoming was obtained from the analysis and from the literature respectively. The corresponding Years of Life Lost (YLL) was derived for each of the countries, following the methodology outlined in the latest global burden of diseases report [9], which applies a standard loss function specifying the years of life lost due to death at a specific age. The standard loss function is based on projected frontier period life expectancy at birth for Japan and South Korea in the year 2050 estimated at 91.9 years and is not discounted [9]. Thus, we defined the YLL due to SBE in each country as 91.9 years minus the mean age at the time of envenoming. The mean ages of SBE were not available for all countries included in our analysis, but were reported for Chad at 25.2 years, Niger at 29 years, Nigeria at 26 years and Mali at 28 years [10,11,12,13]. So, since SBE consistently occurs in victims at a mean age in the late twenties, we made the simplifying assumption that SBE occurs in the 25–29 year age bracket and applied the standard loss function that corresponds to the this age bracket for all countries in our analysis, which is 64.6 years [9]. We then multiplied the number of SBE-related deaths in each country (Table 1, column 2) by 64.6 years to calculate the YLL. Similarly, the Years of Life Lived with Disability (YLD) were estimated by multiplying the number of amputations (Table 1, column 3) by the respective disability weight of 0.13 and applying this disability weight for the remainder of undiscounted local life expectancy [9,14]. In this age group, the remaining local life expectancies for the 16 countries ranged from 37 years in Sierra-Leone to 45 years in Ghana and Senegal (Table 1, column 4). The sum of YLL and YLD then defined the total DALY burden for each country.

**Table 1:** Snakebite burden in Western Africa.

Country	# Deaths (95% Confidence Interval)	#Amputations (95% Confidence Interval)	Local remaining life expectancy at time of bite [i,(years)]	DALYs from YLL [ii]	DALYs from YLD [iii]	Total DALYs (= DALYs from YLL + DALYs from YLD)
Benin	117(93–142)	143(90–244)	44	7558 (6008–9173)	818(515–1396)	8376 (6523–10569)
Burkina Faso	299(236–365)	352(208–600)	43	19315 (15246–23579)	1968(1163–3354)	21283 (16409–26933)
Cameroun	263(208–320)	319(198–544)	41	16990(13437–20672)	1700(1055–2900)	18690 (14492–23572)
Chad	198(156–240)	234(140–399)	38	12791(10078–15504)	1156(692–1971)	13947 (10770–17475)
Cote d’Ivoire	264(209–320)	322(203–550)	39	17054(13501–20672)	1633(1029–2789)	18687(14530–23461)
Gambia	31(24–38)	37(22–63)	44	2003(1550–2455)	212(126–360)	2215 (1676–2815)
Ghana	310(246–376)	379(240–648)	45	20026(15892–24290)	2217(1404–3791)	22243 (17296–28081)
Guinea Bissau	24(19–29)	28(17–48)	41	1550(1227–1873)	149(91–256)	1699 (1318–2129)
Guinea Conakry	159(126–193)	192(118–327)	43	10271 (8140–12468)	1073(660–1828)	11344 (8800–14296)
Liberia	37(30–45)	47(31–81)	44	2390(1938–2907)	269(177–463)	2659 (2115–3370)
Mali	238(188–289)	284(172–484)	43	15375(12145–18669)	1588(961–2706)	16963 (13106–21375)
Niger	264(208–322)	311(183–529)	44	17054(13437–20801)	1779(1047–3026)	18833 (14484–23827)
Nigeria	1927(1529–2333)	2368(1506–4043)	41	124484 (98773–150712)	12621(8027–21549)	137105 (106800–172261)
Senegal	192(152–232)	232(145–397)	45	12430(9819–14987)	1357(848–2322)	13787 (10667–17309)
Sierra Leone	92(73–112)	111(69–190)	37	5943(4716–7235)	534(332–914)	6477 (5048–8149)
Togo	78(62–95)	94(57–160)	43	5039(4005–6137)	525(319–894)	5564 (4324–7031)
Total	4494(3557–5450)	5454(3398–9306)	Not applicable	290,275 (229,911–352,135)	29599(18446–50519)	319874(248357–402654)

^i.^ Local remaining life expectancies in years at time of bite, i.e., for those aged 25–29 years

^ii.^ Product of standard frontier loss of function for those aged 25–29 years (YLL = 64.6 years) and number of deaths (in DALYs)

^iii.^ Product of Years of Life Lived with disability (YLD) [= local remaining life expectancies (i)] and disability weight for amputation (0.13) and number of amputations

## Results

Data on mortality and amputations obtained from Chippaux, 2011 [4] are presented in Table 1. The resulting burden was subsequently derived as described above and compared to other NTDs reported in the Global Burden of Diseases (GBD). In sum, SBE is associated with 319,874 DALYs annually (95% Confidence Interval: 248,357–402,654 DALYs) in the 16 West African countries included in the analysis. Most of the public health burden is due to early mortality, with YLL accounting for 290,275 DALYs (95%CI: 229,911–352,135 DALYs) and YLD accounting for 29,599 DALYs (18,446–50,519 DALYs). The highest local public health burden associated with SBE is estimated for Nigeria, at 137,105 DALYs or 43% of the total burden, followed by Ghana 22,243 DALYs, Burkina Faso 21,283 DALYs, Niger 18,833 DALYs and Cameroun 18,690 DALYs. The lowest public health burden is estimated for Guinea Bissau at 1,699 DALYs or 0.5% of the total burden [Table 1].

### Scenario analysis

Using sub-regional level alternative data that reported low and high estimates of 1504 and 18654 annual snakebite deaths for WA by Kasturiratne et al 2008 [6] yielded burden of YLL from SBE deaths of 97,158 DALYs and 1,205,048 DALYs for low and high estimates respectively. The derived high estimate is 3.77 times higher than that obtained using data from Chippaux 2011 [4].

Similarly, using recent alternative estimates of annual snakebite deaths reported in a WHO document for Benin Republic 650, Burkina Faso 200 and Togo 199 yielded alternative YLL values of 41,990 DALYs, 12,920 DALYs and 12,855 DALYs for those countries respectively [15,16].

## Discussion

**Fig 1:**
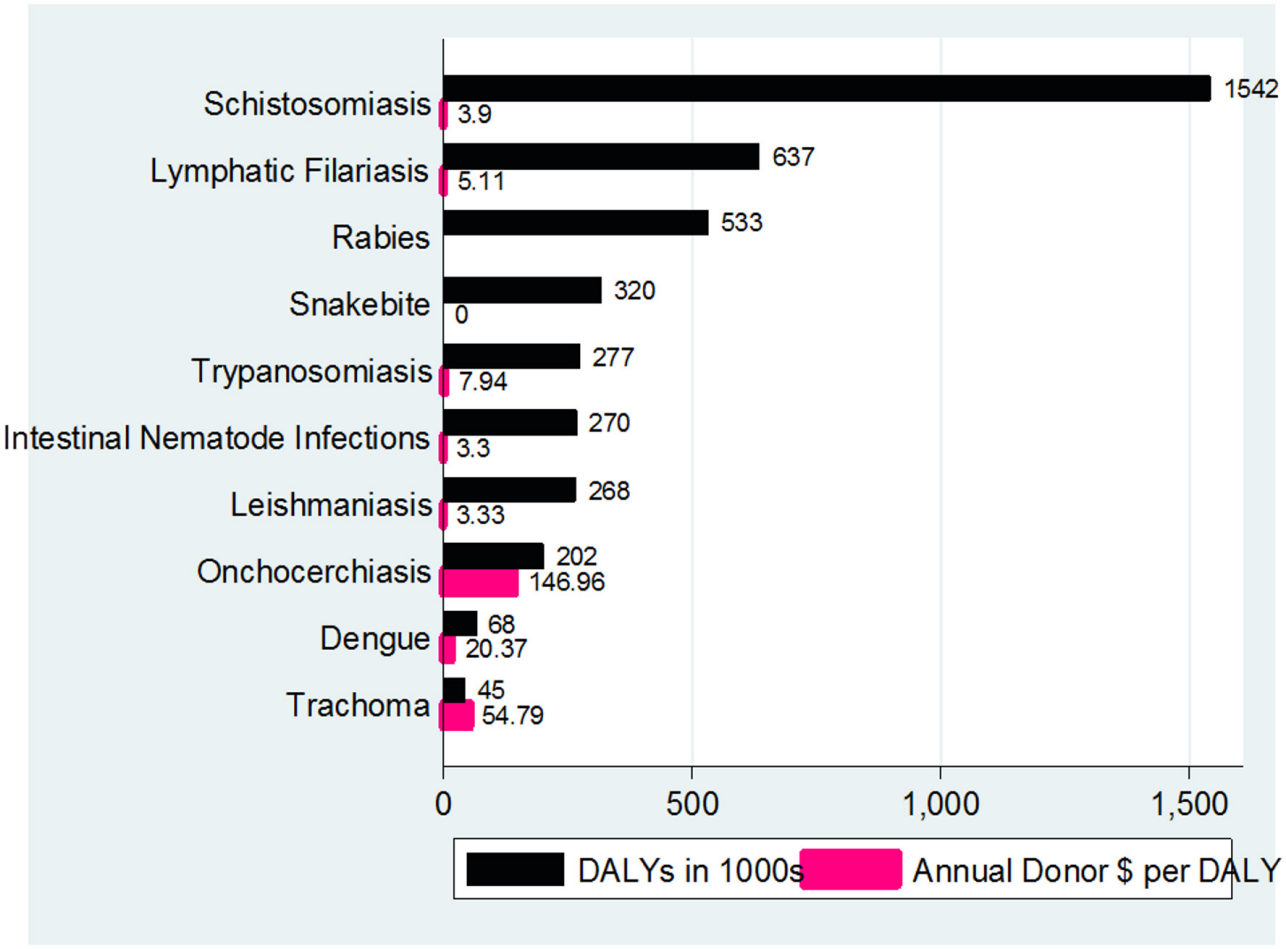
Burden and donor funding of ten neglected conditions in 16 countries in West Africa*. * The Annual Donor $ per DALY for Rabies is not available.

In the current reappraisal of data from WA, SBE accounted for 320,000 DALYs although using higher mortality estimates the YLL could be as high as 1.2 million DALYs [6]. Our estimate of 0.32 million DALYs is higher than the worldwide burden estimated for Buruli ulcer, Echinococcosis, Leprosy, Trachoma, Yaws and Yellow Fever. The estimate is also higher than the burden of African Trypanosomiasis, Leishmaniasis and Onchocerciasis within the 16 countries in WA region [17]. It is also higher than that of Podoconiosis the only other non-communicable disease in the expanded WHO NTD list. Compared to NTDs reported in the Global Health Estimates (GHE) for 2012, SBE has the fourth highest burden in the 16 WA countries, ranking below Schistosomiasis, Lymphatic Filariasis and Rabies [17] (Fig 1). Despite these estimates, SBE remains under-recognized. The resources allocated are not commensurate with its burden. In a study that evaluated funding for developing world health from 42 major donors (comprising industrialized countries 23, international financial institutions 5, multinational pharmaceutical companies 6 and philanthropic foundations 8), annual donor dollar direct funding for 8 of the ten NTDs (Fig 1) ranged from $3.30 per DALY for Intestinal Nematode Infections to $146.96 per DALY for Onchocerchiasis [18]. There is no evidence that any amount was provided for SBE by these donors during the period of the survey.

The difference in burden estimates from the two studies might have arisen from their methodologic approaches [4,6]. The SBE data reported by Chippaux 2011 [4] was obtained using a meta-analytic approach as described above. In contrast the study of Kasturiratne et al 2008 [6] modeled data from electronic databases, indexed and grey literature from 1985. They provided lowest and highest SBE estimates and rates when more than one source was available from a country. This led to a very wide range and imprecise estimates. Furthermore, country-level estimates were not provided and data from Chad, Ghana, Guinea-Bissau, Liberia, Nigeria and Sierra-Leone were not used to derive the mortality estimates for the WA sub-region. They extrapolated from data in adjacent countries, ignoring the geographical variations in snake-bite incidence. Both studies have common limitations. The authors did not choose the survey sites, studied variables and analytical strategies of the data. Most of the collected data were incomplete and spotty, resulting in a questionable representativeness. Nevertheless, some extrapolations were corroborated by national health statistics of some countries, such as in Benin [19].

Disability Adjusted Life Years (DALYs) are the sum of two components: years of life lost (YLLs) and years lived with disability (YLDs). The DALY represents one of the few metrics available that could estimate acute and chronic effects and allow for comparison of significance of burden of several conditions. With a few exceptions, notably Rabies with nearly 100% mortality, most of the NTDs currently listed by the World Health Organization (WHO) and those on the expanded list are disablers rather than killers. In contrast SBE is both a disabler and killer with DALYs accruing from both components. In WA, SBE is an important killer and most of the DALYs (over 90%) accrued from early deaths. These deaths are partially driven by envenoming from saw-scaled or carpet vipers (Genus *Echis*) which cause a high mortality of about 12–20% without antivenom therapy. However, our analysis has been conservative given only amputation was used as the main disability. Several important but rare sequelae (e.g., blindness, malignant ulcers, fetal loss, cognitive and pyschological impairment) were not considered. In WA the frequency of venom ophthalmia and blindness from cobra spits is <0.01% although blindness rarely may result from carpet viper induced ocular bleeding [20,21]. We have also observed 1 case of fetal loss out of 1800 SBE cases or <0.1% but no reports of cognitive/psychological impairment have been made from WA in contrast to Asia [22,23]. This underestimates the total burden and the contribution of DALYs accrued from YLD.

Globally, the public health significance of SBE is generally neglected and underappreciated. It is not among the WHO’s 17 major NTDs although it is mentioned among the ‘other neglected conditions’ (http://www.who.int/neglected_diseases/diseases/en/. However, there is no official WHO program for its prevention or treatment. For the first time, the GBD 2010 provided disease burden estimates for these ‘other NTDs’, i.e., amoebiasis, cryptosporidiosis, trichomoniasis, scabies, fungal skin infections, and venomous animal contact including snakebite, although they are not listed under the NTD and Malaria category. Out of the approximately 48 million DALYs ascribed to both groups of NTDs, venomous animal contact was projected to account for 2.72 million DALYs in the GBD 2010 [24,25]. Interestingly, the annual deaths from SBE in India alone was estimated at 45,900 in the rigorously conducted Million Death Study [26]. While there may be certain minor differences between WA and India, using the approach in this study will translate to 2.97 million DALYs from SBE related YLL in India alone. Thus, estimates from WA and India when combined with the burden from Latin America, Papua New Guinea, the rest of Africa and Asia would be very substantial and much more than the current gross underestimation. Indeed, global burden, using reported high mortality estimates of 93,945 annual deaths worldwide by Kasturiratne et al [6], would result in 6.07 million DALYs.

Effective antivenom therapy has been shown to prevent death from SBE by at least 75% and is a very cost-effective intervention with an incremental cost-effectiveness ratio of $100/DALY averted [8,27]. With expanded access to appropriate and affordable antivenom therapy, the burden of SBE will be considerably curtailed. About $33.61 million (95% Confidence Intervals: $25.85-$43.03 million) will be required annually to control SBE in WA.

This analysis is subject to a number of limitations, including data scarcity, variability and inherent difficulties in accurately estimating the number of incident cases reported in SBE studies. We used the approach adopted by WHO in 2012 and the GBD 2010 in computing DALYs, i.e., with a time discount rate of 0% and no age-weighting [9,24,25]. This is now the standard way to assess disease burden but compared to the previous method it leads to a substantial increase in the absolute number of DALYs lost and a relative increase in the share of DALYs at the extremes of life.

In conclusion, SBE is a major public health problem with a burden higher than that of most other NTDs in the WA sub-region. Commensurate efforts and funding compared to its burden should be made available for control globally and in the sub-region.

## Supporting Information

S1 Annex Guide to meta-analysis estimates. (DOC)
