# Supplementary material for: Snakebite is Under Appreciated: Appraisal of Burden from West Africa
Source: PLoS Negl Trop Dis. 2015 Sep 23;9(9):e0004088. doi: 10.1371/journal.pntd.0004088 (PMC4580425; doi:10.1371/journal.pntd.0004088)
Supplement: S1 Annex — (DOC) [file pntd.0004088.s001.doc]

**S1_Annex**

**Guide To Meta-analysis Estimates**

The search for epidemiological studies on snakebites in Africa was performed through a systematic interrogation of Medline with the following words: Africa AND snake* AND [envenom* OR antiven*]. The references of the papers were searched to find publications in non-indexed journals, thesis and conference proceedings, as well as available clinical reports and grey literature. This survey was completed by systematic searching of non-indexed tropical medicine journals and congress proceedings in English, French, Italian, Spanish, Portuguese and German for the last 40 years.

The data were stratified according to a) their source (case reporting in the national health information system, records from health centers and household surveys), b) into two broad geographical regions (West Africa, from Senegal to Nigeria, and Central, East and Southern Africa, the Somali Cameroon and South Africa), and c) urban versus rural locations. When the source was the records of the health centers, the incidence has been calculated based on the population of hospital catchment area, either specified by the authors or estimated using demographic information available. The localities of over 500,000 inhabitants were considered urban areas. All data was analyzed using the software Comprehensive Meta Analysis v. 2·2·050 software (Biostat®, Englewood, NJ, USA) through the random-effects model [1].

The demographic data to assess the incidence and mortality observed for each study were obtained from national documents and those of the United Nations for the corresponding years [2].

This yielded an average incidence for different parts of Africa. For this study, and based on all the local studies considered in West Africa, it was determined by meta-analysis that:

1. the average annual incidence in rural areas was 47 [39-55; CI=95%] snakebites per 100,000 inhabitants and in urban areas it was 6.4 [5-7.7] per 100,000 inhabitants [1]. To derive the total number of snakebites the incidence of snakebites were applied to the respective population of each country in the study taking into account the rural and urban distribution. These were then combined to give the total annual number of bites.
2. the annual rural mortality was 2.23 [1.75-2.72] per 100,000 inhabitants and urban mortality was 0.15 [0.14-0.16] per 100,000 inhabitants [1]. To derive the total number of snakebite deaths the mortality were applied to the respective population of each country in the study taking into account the rural and urban distribution. These were then combined to give the total mortality.
3. the rate of amputations was 3 [1.9-4.7] % per envenoming. This was also applied to total annual snakebites in each country as derived in (i) above.

The numbers of snakebite deaths and amputations derived from (ii) and (iii) above are provided for each country in Table 1 of the article.

References

1. Chippaux J-P. Estimate of the burden of snakebites in sub-Saharan Africa: A meta-analytic approach. Toxicon 2011; doi:10.1016/j.toxicon.2010.12.022
2. www.un.org/esa/population and <http://unstats.un.org/unsd/demographic/> (accessed on 17/8/2015)
